# Conduction of Electrical Current to and Through the Human Body: A Review

**Published:** 2009-10-12

**Authors:** Raymond M. Fish, Leslie A. Geddes

**Affiliations:** ^a^ Bioacoustics Research Lab & Department of Surgery, University of Illinois at Urbana-Champaign; ^b^ Weldon School of Biomedical Engineering, Purdue University, W Lafayette, Ind

## Abstract

**Objective:** The objective of this article is to explain ways in which electric current is conducted to and through the human body and how this influences the nature of injuries. **Methods:** This multidisciplinary topic is explained by first reviewing electrical and pathophysiological principles. There are discussions of how electric current is conducted through the body via air, water, earth, and man-made conductive materials. There are also discussions of skin resistance (impedance), internal body resistance, current path through the body, the let-go phenomenon, skin breakdown, electrical stimulation of skeletal muscles and nerves, cardiac dysrhythmias and arrest, and electric shock drowning. After the review of basic principles, a number of clinically relevant examples of accident mechanisms and their medical effects are discussed. Topics related to high-voltage burns include ground faults, ground potential gradient, step and touch potentials, arcs, and lightning. **Results:** The practicing physician will have a better understanding of electrical mechanisms of injury and their expected clinical effects. **Conclusions:** There are a variety of types of electrical contact, each with important characteristics. Understanding how electric current reaches and travels through the body can help the clinician understand how and why specific accidents occur and what medical and surgical problems may be expected.

This article explains ways in which electric current is conducted to and through the human body and how this influences the nature of injuries. This multidisciplinary topic is explained in part A by first reviewing electrical and pathophysiological principles, and later in part B by considering specific types of accidents. There are discussions of how electric current is conducted through the body via air, water, earth, and man-made conductive materials. There are discussions of skin resistance (impedance), internal body resistance, current path through the body, the let-go phenomenon, skin breakdown, electrical stimulation of skeletal muscles and nerves, cardiac dysrhythmias and arrest, and electric shock drowning. After the review of basic principles, a number of clinically relevant examples of accident mechanisms and their medical effects are discussed in part B. Topics related to high-voltage burns include ground faults, ground potential gradient, step and touch potentials, arcs, and lightning. Understanding how electric current reaches and travels through the body can help one understand how and why specific accidents occur and what medical and surgical problems may be expected.

## PART A: BASICS OF ELECTRICITY AND HOW IT INTERACTS WITH THE HUMAN BODY

*Electric shock* is defined as a sudden violent response to electric current flow through any part of a person's body. *Electrocution* is death caused by electric shock. *Primary electrical injury* is tissue damage produced directly by electrical current or voltage. Secondary injuries, such as falls, are common. Unless otherwise noted, this article is referring to currents and voltages of 60 (or 50) Hz AC rms. Also, by resistance, we actually mean the magnitude of the impedance. *High voltage* refers to 600 V or more AC rms.

## Very small amounts of electric current result in major physiological effects

*Current* refers to the amount of electricity (electrons or ions) flowing per second. Current is measured in amperes or milliamperes (1 mA=1/1000 of an ampere). The amount of electric current that flows through the body determines various effects of an electric shock. As listed in Table 1, various amounts of current produce certain effects. Most current-related effects result from heating of tissues and stimulation of muscles and nerves. Stimulation of nerves and muscles can result in problems ranging from a fall due to recoil from pain to respiratory or cardiac arrest. Relatively small amounts of current are needed to cause physiological effects. As shown in the table, it takes a thousand times more current to trip a 20-A circuit breaker than it takes to cause respiratory arrest.

## Skin resistance protects the body from electricity

The body has *resistance* to current flow. More than 99% of the body's resistance to electric current flow is at the skin. Resistance is measured in ohms. A calloused, dry hand may have more than 100,000 Ω because of a thick outer layer of dead cells in the stratum corneum. The internal body resistance is about 300 Ω, being related to the wet, relatively salty tissues beneath the skin. The skin resistance can be effectively bypassed if there is skin breakdown from high voltage, a cut, a deep abrasion, or immersion in water (Table 2). The skin acts like an electrical device such as a capacitor in that it allows more current to flow if a voltage is changing rapidly. A rapidly changing voltage will be applied to the palm and fingers of one's hand if it is holding a metal tool that suddenly touches a voltage source. This type of contact will give a much greater current amplitude in the body than would otherwise occur.^2^

## Voltage

Voltage can be thought of as the force that pushes electric current through the body. Depending on the resistance, a certain amount of current will flow for any given voltage. *It is the current that determines physiological effects*. Nevertheless, voltage does influence the outcome of an electric shock in a number of ways, as discussed below.

## Skin breakdown

At 500 V or more, high resistance in the outer layer of the skin breaks down.^3^ This lowers the body's resistance to current flow greatly. The result is an increase in the amount of current that flows with any given voltage. Areas of skin breakdown are sometimes pinhead-sized wounds that can be easily overlooked. They are often a sign that a large amount of current could enter the body. This current can be expected to result in deep tissue injury to muscles, nerves, and other structures. This is one reason why there is often significant deep tissue injury little in the way of skin burns with high-voltage injuries.

## Electroporation

Electroporation (cell membrane damage) is due to the application of a large voltage across a length of tissue. This would occur with 20,000 V from hand to hand. Electroporation would also occur with 120 V with the end of a power cord in a child's mouth. In this situation, the voltage is not high, but the volts per inch of tissue is the same as in the case when high voltage is applied from hand to hand or head to foot. As a result of electroporation, even brief contact can result in severe muscle and other tissue injuries. Electroporation is another reason for the occurrence of deep tissue injury.

## Heating

Other things being equal, the heat energy delivered to tissues is proportional to the square of the voltage (increasing the voltage by a factor of 10 increases the heat energy by a factor of 100).

## Alternating and direct current

Membranes of excitable tissues (eg, nerve and muscle cells) will pass current into cells most effectively when an applied voltage is changing. The skin is somewhat similar in that it passes more current when the voltage is changing. Therefore, with alternating current, there is a continuous changing of the voltage, with 60 cycles of voltage change occurring per second. With alternating current, if the current level is high enough, there will be a feeling of electric shock as long as contact is made. If there is enough current, skeletal muscle cells will be stimulated as rapidly as they can respond. This rate is slower than 60 times per second. This will give a tetanic muscle contraction, resulting in the loss of voluntary control of muscle movements. Cardiac muscle cells will receive 60 stimulations per second. If the amplitude of the current is sufficient, ventricular fibrillation will occur. The heart is most sensitive to such stimulation during the “vulnerable period” of the cardiac cycle that occurs during much of the T wave.

In contrast, with direct current, there is a feeling of shock only when the circuit is made or broken unless the voltage is relatively high.^4^ Even if the current amplitude is large, it may not occur during the vulnerable period of the cardiac cycle. With alternating current, a shock duration of longer than 1 cardiac cycle will definitely give stimulation during the vulnerable period.

## How current, voltage, and resistance are related

**Figure 1:**
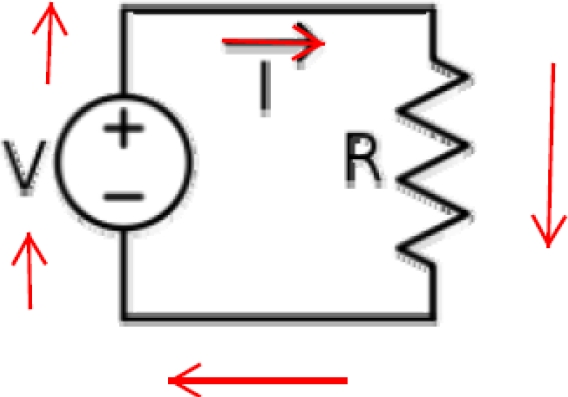
Voltage causes current (*I*) to flow through a given resistance. The somewhat circular current path is referred to as a circuit.

Ohm's law is as follows:

Figure 1 shows a voltage source and a resistor. As an example, a 1000-Ω resistance connected to a 120-V electrical source will have

## Current path(s)

Electricity flows from (at least) one point to another. This is often from one terminal to the other terminal of the voltage source. The connection between the terminals of a voltage source is often referred to as a “load.” The load can be anything that conducts electricity, such as a light bulb, a resistor, or a person. This is shown in Figure 1.

To illustrate some important issues, this circuit model can be applied to a car. For example, the negative terminal of a car battery is connected (“grounded”) to the metal chassis of the car. The positive terminal is connected to a red cable made of individual wires that go to the starter, lights, air conditioner, and other devices. Electric current flows through many parallel paths: the radio, the starter, the lights, and many other current paths. The current in each path depends on the resistance of each device. Disconnecting either the positive or negative terminal of the battery will stop the flow of current, although the other connection is intact.

## Applying the model to the human body

The example of the car makes it easier to understand current flow in the human body. A person receiving an electrical shock will have (at least) 2 contact points to a voltage source, one of which might be the earth ground. If *either* connection is disconnected, no current will flow. The analogy also explains how current flow can go through many somewhat parallel pathways, such as through the nerves, muscles, and bones of the forearm. The amount of current in each automobile appliance or tissue type depends on the resistance of each component.

**Figure 2:**
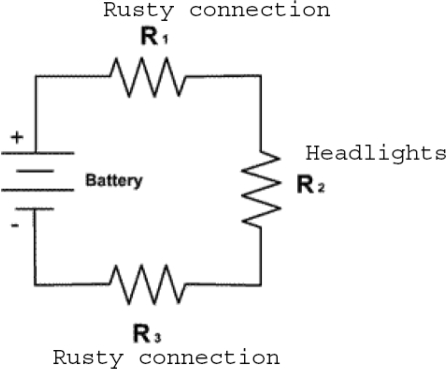
Rusty contacts add resistance to current flow. The headlights are analogous to the internal body resistance, and the rusty connections are similar to skin resistance. Total body resistance is equal to the internal body resistance plus the 2 skin resistances.

Figure 2 takes the model a step further. It shows the battery and headlights on a bicycle. There are rusty connections on both the positive and negative battery terminals. The total resistance the voltage must push current through is that of the 2 rusty connections in addition to the resistance of the headlights. *More resistance results in less current flow*. The rusty connection is analogous to skin resistance, and the headlight is analogous to the internal body resistance. *The total body resistance is equal to the internal body resistance plus the 2 skin* resistances.

**Figure 3:**
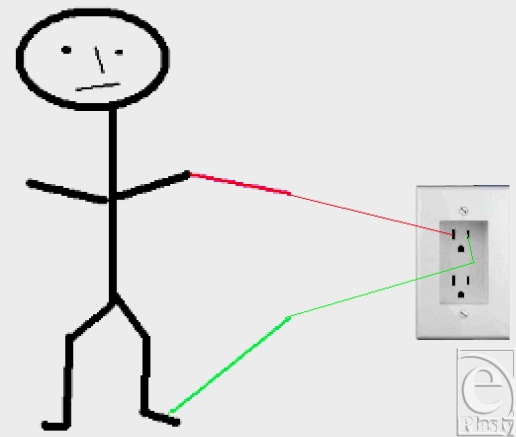
Diagram of a person connected to a voltage source.

Figure 3 shows a person connected to a voltage source. There are connections to the left hand and the left foot. The “total body resistance” of the person is composed of the very low (approximately 300 Ω) internal body resistance plus the 2 skin contact resistances. The skin contact resistance will usually be between 1000 and 100,000 Ω, depending on contact area, moisture, condition of the skin, and other factors. The skin thus provides most of the body's protection from electric current.

## High-voltage contact

High-voltage (≥600 V) contacts sometimes seem paradoxical. A bird comfortably sits on a high-voltage power line. But a person with work boots standing next to a truck is killed on touching the side of the truck because an elevated attachment to the truck was touching a power line. High voltage breaks down electrical insulators, including paint, skin, and most shoes and gloves. Special shoes, gloves, and tools are rated as being protective for certain voltage levels. These items must be tested periodically for (sometimes pinpoint sized) breaks in insulation. Insulation may not be effective if there is moisture or contamination on the surface of the item.

As noted above, current flow requires 2 or more contact points that are at different voltages. Many electrical systems are connected (“grounded”) to the earth. Support structures are often metal and also physically in the ground.

The workman was connected electrically to the power line through the metal parts of his truck. The high voltage (7200 V) was high enough to go through the paint on the truck and his shoes. The bird was not close enough to the ground or anything else to complete the circuit to ground. There are birds with large wingspans that do get electrocuted when they bridge the gap between wires and structures that are at different voltages.

## PART B: TYPES OF ELECTRICAL CONTACT

### Step and touch potentials

The earth (ground) under our feet is usually considered to be at 0 V. Power lines and radio antennas are grounded by connecting them to metal rods driven into the ground. If a person is barefoot on the ground with his or her feet spread apart, there should be 0 V between the 2 feet. This normal state of affairs is disrupted if a conductor from a high-voltage power line reaches the ground or if lightning strikes the ground.

Voltage from overhead power lines can reach the ground in several ways. A line can break or come loose from its insulated supports and make contact with the ground itself or with structures that are themselves connected to the earth. Supporting wires (guy wires) may come loose from their connections near the ground and become energized when they come into contact with a power line. The energized guy wire is then at a high voltage. If the guy wire contacts the ground, the voltage on the earth at and around the contact point is no longer 0 V.

When an energized conductor contacts the ground directly or through a conductor, it is referred to as a ground fault. *The decrease of voltage with distance* from the earth contact point of an energized object is called the *ground potential gradient*. Voltage drops associated with this dissipation of voltage are called ground potentials.

**Figure 4:**
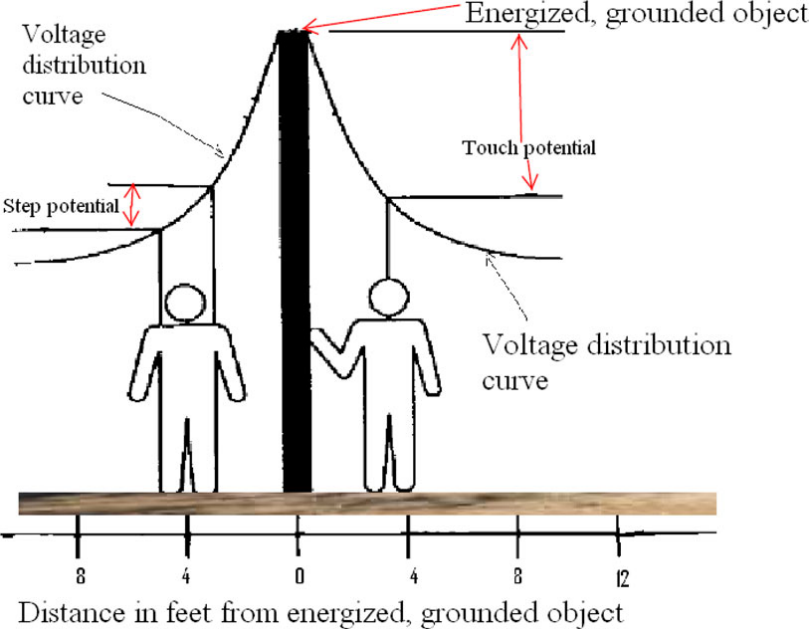
Step and touch potentials. Actual numbers may vary with soil type and moisture as well as other factors.

Figure 4 is a typical voltage-gradient distribution curve. This graph shows that voltage decreases with increasing distance from the grounding object. On the left of the grounded, energized object, there is a voltage difference between the person's 2 feet, referred to as a step potential. On the right, there is a voltage difference between the person's hand and 2 feet, referred to as a touch potential. There is also a step potential between the 2 feet of the person on the right. (Figure 4 and this section are modifications of part of OSHA Regulations [Standards-29 CFR].)

## Flash burn, electric current heating, or both

High-voltage arcs involve passage of electricity through the air. In some cases, the arc does not contact a person. In this situation, there can be serious burns from the heat of the arc (a flash burn). There can also be burns from burning clothing and other substances. Burns can also result from touching objects that are thermally hot but not electrically energized.

High-energy arcs can produce explosion-related shock waves.^5^ The blunt trauma force that results can throw a person, rupture eardrums, and contuse internal organs.

If the arc or an energized conductor does contact the person and electricity flows through him or her, there can be injury from the electric current flow through the body in addition to the injury mechanisms mentioned above.

It is of clinical importance to determine whether a high-voltage injury involved electric current flow through the body. Current flow through the body due to high voltage can result in conditions that must be watched over time. These conditions include myoglobinuria, coagulopathy, and compartment syndromes. Several clinical and electric contact–related issues can help one determine whether there has been current flow through the body. First, electric current flow through the body requires at least 2 contact points. With high voltage, these are generally full-thickness burns. They can be pinhead sized and are sometimes multiple due to sparking. If a conductor such as a piece of wire contacted the skin, there may be a burn injury with the shape of the object contacted.

A flash burn with no current through the body, in contrast, tends to be diffuse and relatively uniform. Flash burns are *sometimes* less than full thickness, whereas a high-voltage contact burn will be full thickness.

## So-called entry and exit wounds

There are often just 2 contact burns that are generally referred to as entrance and exit wounds. These terms relate to the fact that electric current comes from a voltage source, enters the body at one point, flows through the body to the other contact point, where it exits the body, and returns to the voltage source (or a ground). This terminology is somewhat confusing if one considers that alternating current changes direction many times per second. The terminology may also be misleading because it reminds one of bullet wounds that sometimes have small entry and larger exit wounds. With electrical injury, the size of the wound will depend on factors such as the size and shape of the conductor, the geometry of the body part involved, and moisture. The analogy to gunshot wounds is also misleading in that there is not always a bullet exit wound because the bullet remains lodged in the person. Thus, 2 separate third-degree burns suggest current flow through the body. A diffuse, partial-thickness burn does not suggest current flow through the body.

In addition to the contact-related features, there are clinical signs that can help determine whether there was current flow through deep tissues. For example, a high-voltage contact to the hand associated with current flow into the arm would be expected to produce forearm firmness and tenderness. There would be pain with passive and active finger movements, and there may be sensory deficits in the hand.

## Lightning

Lightning typically flashes over the surface of the body, resulting in surprisingly little damage in some persons. Wet skin and the very brief nature of the pulses of electricity encourage the current to travel on the surface of the body. Nevertheless, lightning does sometimes injure persons because of current flow in the body, blunt mechanical force, a blast effect that may rupture eardrums and contuse internal organs, and intense light that can result in cataracts.

## Contact with conductors

### Low voltage (<600 V)

The effects of low-voltage shocks are listed in Table 1. The current levels given vary with the specific current path, duration of contact, the person's weight, height, and body build (especially musculature and bony structures), and other factors. The effects that do occur in any specific case are strongly dependent on several factors related to how contact is made with the source of electricity. These factors include current path, moisture, if there was inability to let go, and the size of the areas of contact.

## Current path

If the current path goes through the chest, continuous tetanic contractions of the chest wall muscles can result in respiratory arrest. Dalziel,^6^ who made measurements on human subjects, relates that currents in excess of 18 mA stimulate the chest muscles so that breathing is stopped during the shock.

Another effect that occurs with a transthoracic current path is ventricular fibrillation. Transthoracic current paths include hand to hand, hand to foot, and front of the chest to the back of the chest. Animal experiments have suggested that the ventricular fibrillation threshold is inversely proportional to the square root of the duration of current flow.

## The let-go phenomenon for low (<600 V) contact

A factor that makes a large difference in the injury sustained in low-voltage shocks is the inability to let go. The amount of current in the arm that will cause the hand to involuntarily grip strongly is referred to as the let-go current.^7^ If a person's fingers are wrapped around a large cable or energized vacuum cleaner handle, for example, most adults will be able to let go with a current of less than 6 mA. At 22 mA, more than 99% of adults will not be able to let go. The pain associated with the let-go current is so severe that young, motivated volunteers could tolerate it for only a few seconds.^7^ With current flow in the forearm, the muscles of flexion and extension are both stimulated. However, the muscles of flexion are stronger, making the person unable to voluntarily let go. Nearly all cases of inability to let go involve alternating current. Alternating current repetitively stimulates nerves and muscles, resulting in a tetanic (sustained) contraction that lasts as long as the contact is continued. If this leads to the subject tightening his or her grip on a conductor, the result is continued electric current flow through the person and lowered contact resistance.^8^

With alternating current, there is a feeling of electric shock as long as contact is made. In contrast, with direct current, there is only a feeling of shock when the circuit is made or broken. While the contact is maintained, there is no sensation of shock. Below 300 mA DC rms, there is no let-go phenomenon because the hand is not involuntarily clamped. There is a feeling of warmth while the current travels through the arm. Making or breaking the circuit leads to painful unpleasant shocks. Above 300 mA, letting go may be impossible.^4^ The threshold for ventricular fibrillation for direct current shocks longer than 2 seconds is 150 mA as compared with 50 mA for 60-Hz shocks; for shocks shorter than 0.2 seconds, the threshold is the same as that for 60-HZ shocks, that is, approximately 500 mA.^4^

Heating power is also increased when a person cannot let go. This is because a firm grip increases the area of skin effectively in contact with the conductors. Additionally, highly conductive sweat accumulates between the skin and conductors over time. Both of these factors lower the contact resistance, which increases the amount of current flow. In addition, the heating is greater because the duration of the contact is often several minutes in comparison with the fraction of a second that it takes to withdraw from a painful stimulus.

Being unable to let go results in more current for a longer period of time. This will increase damage due to heating of muscle and nerves. There will also be an increase in pain and the incidence of respiratory and cardiac arrest. There can also be shoulder dislocation with associated tendon and ligament injury, as well as bony fractures in the area of the shoulders.

## The let-go phenomenon for high (>600 V) contact

Several different outcomes may occur when a person grasps a conductor giving 10 kV AC hand-to-hand voltage. It takes over 0.5 seconds of such contact before most of the distal forearm cells are heat damaged. However, within 10 to 100 milliseconds, muscles in the current path will strongly contract. The person may be stimulated to grasp the conductor more tightly, making a stronger mechanical contact. Or, the person may be propelled away from the contact. Which of these events occurs depends on the position of the hand relative to the conductor. Most eyewitnesses report the victims being propelled from the conductor, possibly because of generalized muscle contractions. The time of contact is estimated to be about 100 milliseconds or less in such cases.^9^^(p57)^

## Immersion contact: Electric shock drowning

### Clinical issues

Drowning and near drowning can result from electricity in the water. Conditions requiring treatment of near drowning caused by electricity are mostly the same as conditions related to nonelectrical near drowning. These conditions include myoglobin elevations that can result in renal failure (detected by creatine kinase [CPK] elevations and urine examination), adult respiratory distress syndrome, hypothermia, hypoxia, electrolyte abnormalities, and arrhythmias that include ventricular tachycardia and ventricular fibrillation. Creatine kinase and myoglobin levels *in nonelectrical* near-drowning events are thought to be due to a violent struggle, along with sometimes prolonged hypoxia and electrolyte imbalances. Electricity in the water can stimulate muscles strongly enough to give a person severe muscle pain during and after his or her near-drowning experience. This would further increase CPK and myoglobin levels beyond those that would result from a nonelectrical near drowning Table 3. Creatine kinase levels sometimes rise for a day or more, being influenced by treatment given, continued hypoxia or hypotension, and other conditions that might influence continuing necrosis of tissue.

## Effects of electric current

Many of the determinations of electrical current effects in humans were made by Dalziel.^10^ For any given effect, such as tetanic muscle contractions, there is a range of current levels that produce the effect due to individual subject differences. For example, the current needed to cause tetanic muscle contractions in the forearm (the “let-go” current) can be from 6 to 24 mA (60-Hz AC rms) depending on the subject. Therefore, current levels listed in publications may be maximum, average, or minimum levels, depending on the issues being discussed. For safety issues, near-minimum values are often appropriate.

As listed in Table 4, Dalziel^7^ found that 10 mA would cause tetanic muscle contractions and thus loss of muscle control. In addition, Smoot and Bentel^12^ found that 10 mA of current was enough to cause loss of muscle control in water. They carried out measurements in salt water and did not report voltages that were applied.

## Total body resistance in water

The total body resistance from hand to foot in water is considered to be 300 Ω when considering safety precautions.^13^–^15^ Smoot^11^,^16^ measured a total body resistance of 400 Ω with immersion. Much of this is due to the internal body resistance. Thus, immersion eliminates most of the skin resistance.

Salt water is very conductive compared with the human body, making electric shock drowning in salt water relatively rare. This is because much of the electric current is shunted around the outside of the body.

If there is a voltage difference, for example, between one arm and the other, then electric current will flow through the body. The amount of current is equal to the voltage divided by the total body resistance.

## How much voltage in the water can be lethal?

Table 1 lists the amounts of current needed to cause ventricular fibrillation and other fatal conditions. The total body resistance in water is of 300 Ω. Thus, the current needed and the resistance it must experience are known. It is therefore possible to calculate the voltage needed. For ventricular fibrillation, the calculation is as follows:

Required voltage = Current × Resistance

For causing ventricular fibrillation, the required voltage is as follows:

Voltage = 100 mA × 300Ω = 30 V

Figures for other mechanisms of death are listed in Table 4.

Water-related electrical contact often occurs in 2 ways. These mechanisms can happen in bathtubs, swimming pools, and lakes. The first mechanism of contact involves a person in water reaching out of the water and contacting an energized conductive object. For example, a person is well-grounded by sitting in a bathtub. The resistance of the contact with his hand touching an energized object outside of the tub may be high enough to protect him or her, especially if his or her hand is not wet and the area of contact is small.

The second contact mechanism involves a person in the water being in an electric field because of an energized conductor that is in the water. For example, an electric heater connected to the hot wire of the 120 V AC outlet falls in the water. The grounded drain is close to the person's shoulders, whereas the heater is near his or her feet. This gives a voltage difference of 120 V AC from shoulders to the feet. With a total body resistance of 300 Ω, 360 mA flows, more than 3 times the amount needed to give ventricular fibrillation.

In lakes, ponds, and other water bodies, an electrical power source can generate current into the water. The location of voltages in the water can be measured. Voltages may be present in the water because of the hull of a boat connected to an on-shore power source is energized. Voltages may also be present in the water because of energized conductors in the water that release electrical current into the water.

An electric gradient (or field) can exist that is analogous to the situation described above for step and touch potentials. The situation is more complex to analyze in the water because a person in the water assumes different postures and orientations in 3 dimensions (up, down, and sideways—north, south, east, and west). The transthoracic and translimb voltages will vary as the person moves in relation to the orientation (direction) of the electric field.

## Measurements of loss of muscle control in water

**Figure 5:**
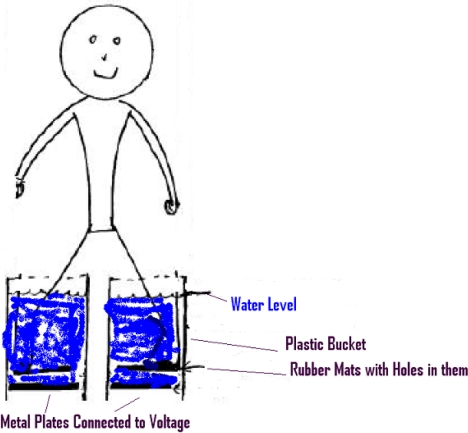
Measurement setup for voltage and current in water.

Measurements similar to those of Smoot and Bentel^12^ were done with approval of the institutional review board of University of Illinois in Urbana-Champaign. Metal plates were placed inside rubber containers. The metal plates were flat on the bottoms of the containers. A rubber mat with holes was placed on top of each metal plate. An (isolated) power source ground wire was connected to one plate, and a 60-Hz AC voltage from the power source was connected to the other plate. The subject stood with 1 foot on each rubber mat, as shown in Figure 5. Thus, the subject's contact with electric current was primarily through the water contacting the feet through the holes and also through water contacting the legs higher up. This foot-to-foot current path simulated the hand-to-hand and hand-to foot situations that can occur with swimmers in water. This setup minimized current flow through the chest. The study involved just 1 subject.

Fresh (not salt) water with conductivity of 320 µmho/cm filled each bucket to a level near the hip. It was found that electrically induced muscle contractions were greatly modified by leg position in the water.

Initial testing has shown that with 3.05 V (60-Hz AC rms) applied between the plates, a current of 8.65 mA flowed, resulting in involuntary flexion of the knee to 90°. This flexion could not be overcome with voluntary effort. The knee could be flexed further voluntarily, but it would not straighten beyond 90° of flexion. The involuntary keen flexion occurred when the leg was lifted (by hip flexion) so that the thigh was horizontal and the knee was at the water level. This is similar to the situation when one is swimming. Muscle control was gradually regained when the foot was lowered to the bottom of the bucket (by hip extension to the neutral position) and the leg became vertical. Total body resistance would be calculated as follows:

At 4.05 V, a current of 12.6 mA flowed. The knee was flexed to 135°, meaning that the heel was near the buttocks. This could not be overcome with voluntary effort. Again, this occurred when the leg was lifted so that the knee was at the water level, similar to the situation when one is swimming. Lesser impairment of muscle control was noted at other leg positions. Muscle control was gradually regained when the foot was lowered to the bottom of the bucket and the leg became vertical. The resistance would be 4.05 V/12.6 mA = 332 Ω.

The current levels measured in these experiments are consistent with those reported by Dalziel,^7^ Smoot,^11^ and NIOSH,^1^ as listed in Tables 1 and 4. The total resistance of the system (water plus subject) is close to 300 Ω, so often mentioned in literature.

## CONCLUSION

There are a variety of types of electrical contact, each with important characteristics. Understanding how electric current reaches and travels through the body can help the clinician understand how and why specific accidents occurred and what medical and surgical problems may be expected.

## Figures and Tables

**Table 1:** Estimated effects of 60 Hz AC currents*

1 mA	Barely perceptible
16 mA	Maximum current an average man can grasp and “let go”
20 mA	Paralysis of respiratory muscles
100 mA	Ventricular fibrillation threshold
2 A	Cardiac standstill and internal organ damage
15/20 A	Common fuse breaker opens circuit†

*From NIOSH.^1^

†Contact with 20 mA of current can be fatal. As a frame of reference, common household circuit breaker may be rated at 15, 20, and 30 A.

**Table 2:** Ways protective skin resistance can be greatly reduced

•	Significant physical skin damage: cuts, abrasions, burns
•	Breakdown of skin at 500 V or more
•	Rapid application of voltage to an area of the skin
•	Immersion in water

**Table 3:** Why immersion in water can be fatal with very low voltages

1	Immersion wets the skin very effectively and greatly lowers skin resistance per unit area
2	Contact area is a large percentage of the entire body surface area
3	Electric current may also enter the body through mucous membranes, such as the mouth and throat
4	The human body is very sensitive to electricity. Very small amounts of current can cause loss of ability to swim, respiratory arrest, and cardiac arrest

**Table 4:** Mechanisms of death in electric shock drowning

Mechanism	Current needed, mA	Voltage needed, V AC
Electrical stimulation of the heart causing ventricular fibrillation	100	30
Tetanic contraction (effectively paralysis) of the muscles of respiration	20	6
Loss of muscle control of the extremities: 16 mA for an average man^1^	16	4.8
Loss of muscle control of the extremities: as little as 10 mA for the most sensitive female^7^,^11^	10	3
